# iCSDB: an integrated database of CRISPR screens

**DOI:** 10.1093/nar/gkaa989

**Published:** 2020-11-02

**Authors:** Ahyoung Choi, Insu Jang, Heewon Han, Min-Seo Kim, Jinhyuk Choi, Jieun Lee, Sung-Yup Cho, Yukyung Jun, Charles Lee, Jaesang Kim, Byungwook Lee, Sanghyuk Lee

**Affiliations:** Department of Bio-Information Science, Ewha Womans University, Seoul 03760, Republic of Korea; Korea Bioinformation Center, Korea Research Institute of Bioscience and Biotechnology, Daejeon 34141, Republic of Korea; Department of Bio-Information Science, Ewha Womans University, Seoul 03760, Republic of Korea; Korea Bioinformation Center, Korea Research Institute of Bioscience and Biotechnology, Daejeon 34141, Republic of Korea; Korea Bioinformation Center, Korea Research Institute of Bioscience and Biotechnology, Daejeon 34141, Republic of Korea; Ewha-JAX Cancer Immunotherapy Research Center, Ewha Womans University, Seoul 03760, Republic of Korea; Department of Biomedical Sciences, Seoul National University College of Medicine, Seoul 03080, Republic of Korea; Ewha-JAX Cancer Immunotherapy Research Center, Ewha Womans University, Seoul 03760, Republic of Korea; The Jackson Laboratory for Genomic Medicine, Farmington, CT 06032, U.S.A; Ewha-JAX Cancer Immunotherapy Research Center, Ewha Womans University, Seoul 03760, Republic of Korea; The Jackson Laboratory for Genomic Medicine, Farmington, CT 06032, U.S.A; Precision Medicine Center, The First Affiliated Hospital of Xi’an Jiaotong University, Xi’an 710061, People’s Republic of China; Ewha-JAX Cancer Immunotherapy Research Center, Ewha Womans University, Seoul 03760, Republic of Korea; Department of Life Science, Ewha Womans University, Seoul 03760, Republic of Korea; Korea Bioinformation Center, Korea Research Institute of Bioscience and Biotechnology, Daejeon 34141, Republic of Korea; Department of Bio-Information Science, Ewha Womans University, Seoul 03760, Republic of Korea; Ewha-JAX Cancer Immunotherapy Research Center, Ewha Womans University, Seoul 03760, Republic of Korea; Department of Life Science, Ewha Womans University, Seoul 03760, Republic of Korea

## Abstract

High-throughput screening based on CRISPR-Cas9 libraries has become an attractive and powerful technique to identify target genes for functional studies. However, accessibility of public data is limited due to the lack of user-friendly utilities and up-to-date resources covering experiments from third parties. Here, we describe iCSDB, an integrated database of CRISPR screening experiments using human cell lines. We compiled two major sources of CRISPR-Cas9 screening: the DepMap portal and BioGRID ORCS. DepMap portal itself is an integrated database that includes three large-scale projects of CRISPR screening. We additionally aggregated CRISPR screens from BioGRID ORCS that is a collection of screening results from PubMed articles. Currently, iCSDB contains 1375 genome-wide screens across 976 human cell lines, covering 28 tissues and 70 cancer types. Importantly, the batch effects from different CRISPR libraries were removed and the screening scores were converted into a single metric to estimate the knockout efficiency. Clinical and molecular information were also integrated to help users to select cell lines of interest readily. Furthermore, we have implemented various interactive tools and viewers to facilitate users to choose, examine and compare the screen results both at the gene and guide RNA levels. iCSDB is available at https://www.kobic.re.kr/icsdb/.

## INTRODUCTION

High-throughput screening (HTS) has become an indispensable tool for functional genomics and drug discovery. Genetic loss-of-function (LoF) experiments are frequently used in HTS to identify targets that confer genetic vulnerability in various diseases. RNA interference (RNAi) screening using small-interfering RNA (siRNA) libraries was the method of choice for LoF experiments during the last decade (1). For example, the Dependency Map (DepMap) project at the Broad Institute (2) used a genome-wide pooled shRNA library to identify and catalog gene essentiality across hundreds of genomically characterized cancer cell lines from the Cancer Cell Line Encyclopedia (CCLE) project (3). Similarly, drug sensitivity was measured in these cell lines for 265 drugs to provide the landscape of pharmacogenomic interactions in cancer (4). Subsequent analysis of combining genetic screening data with drug sensitivity data offered an unprecedented opportunity to decipher the mechanism-of-action of drugs in a genome-wide and unbiased way (5).

CRISPR/Cas9-mediated genome editing that generates the double-strand breaks in the target DNA is rapidly replacing the RNAi method for studying LoF consequences of target genes mainly due to high efficiency (knockout rather than knockdown) and high specificity (i.e. low off-target effect by the usage of relatively long sequences in guide RNA design) (6). Genome-scale CRISPR libraries coupled with the deep sequencing are frequently adopted for functional screens both in negative and positive selections (7). Recently, large-scale LoF screen projects such as the DepMap and Sanger’s GDSC (Genomics of Drug Sensitivity in Cancer) have produced representative data sets of CRISPR-Cas9 screens for the CCLE cell lines (7,8).

Currently, there are several databases in which results of CRISPR screens are aggregated. GenomeCRISPR (9) and PICKLES (10) are two of the earlier databases, but their data contents are already obsolete as important results during the last 3 years are missing. The DepMap portal (https://depmap.org/portal/) is the largest and latest collection of CRISPR screens integrating three large-scale projects (the Broad Achilles screens known as DepMap 20Q2, Sanger CRISPR screens (7) and GeCKO libraries from the Zhang lab (11)). It also supports an elaborate data explorer equipped with advanced analytical tools. BioGRID, the database of protein–protein interactions, opened a new dataset of CRISPR phenotype screens (ORCS) that would serve as a data warehouse for the published CRISPR screen results using human, mouse and fly cell lines (12). Such comprehensive curation efforts are valuable but no further analysis or analytical tools are supported. iCSDB is our own effort to integrate virtually all of the available CRISPR screens featuring many novel analytical tools in a user-friendly web-based environment.

## RESULTS

### Data contents and aggregation

**Figure 1.**
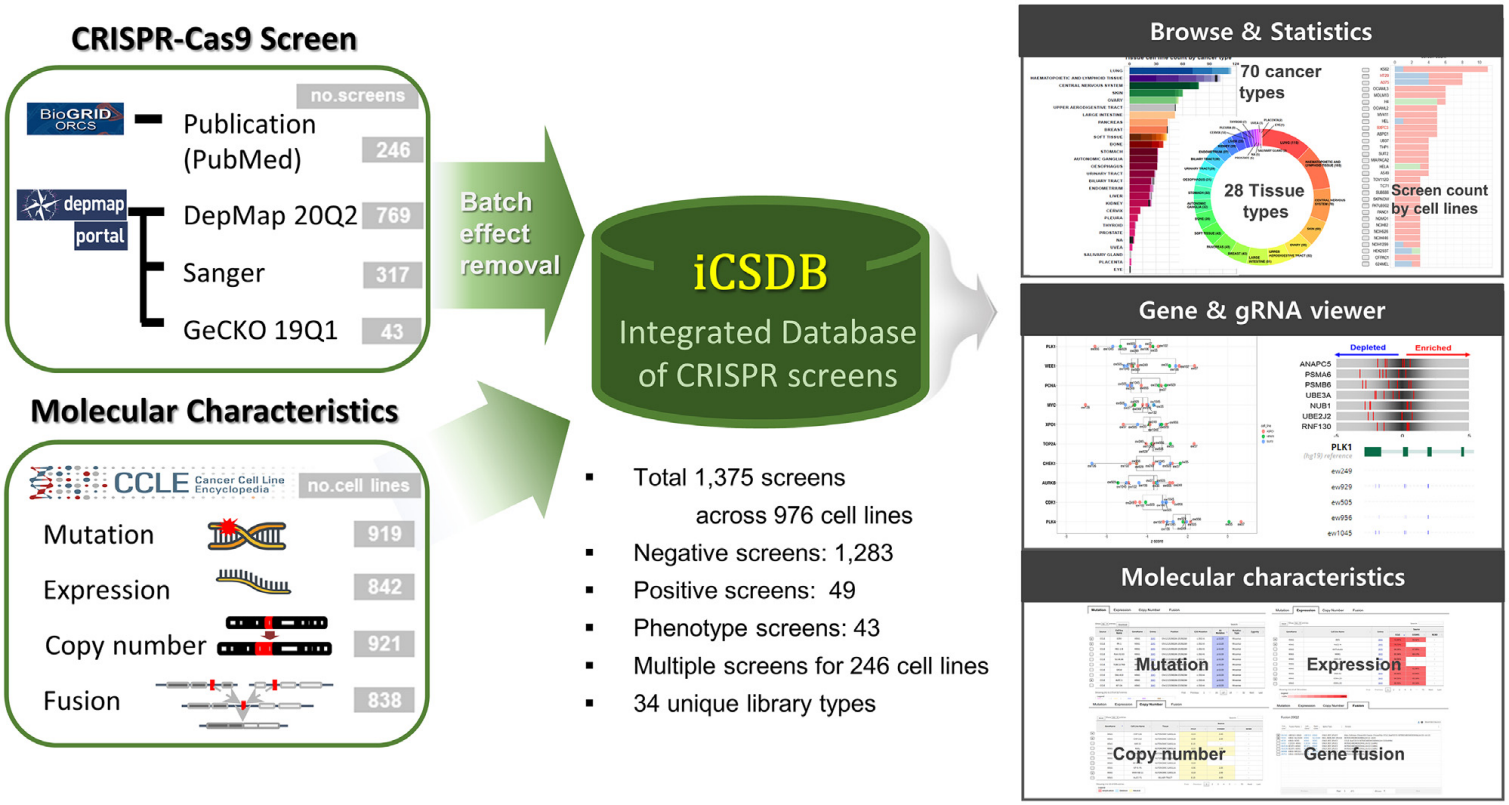
Overview of iCSDB.

The overall contents of iCSDB consist of a collection of genome-wide CRISPR screens, molecular characteristics of cell lines and analytical tools (Figure 1). Molecular characteristics include the mutation, expression, copy number, gene fusion profiles from the CCLE consortium (2019 release) (13).

**Figure 2.**
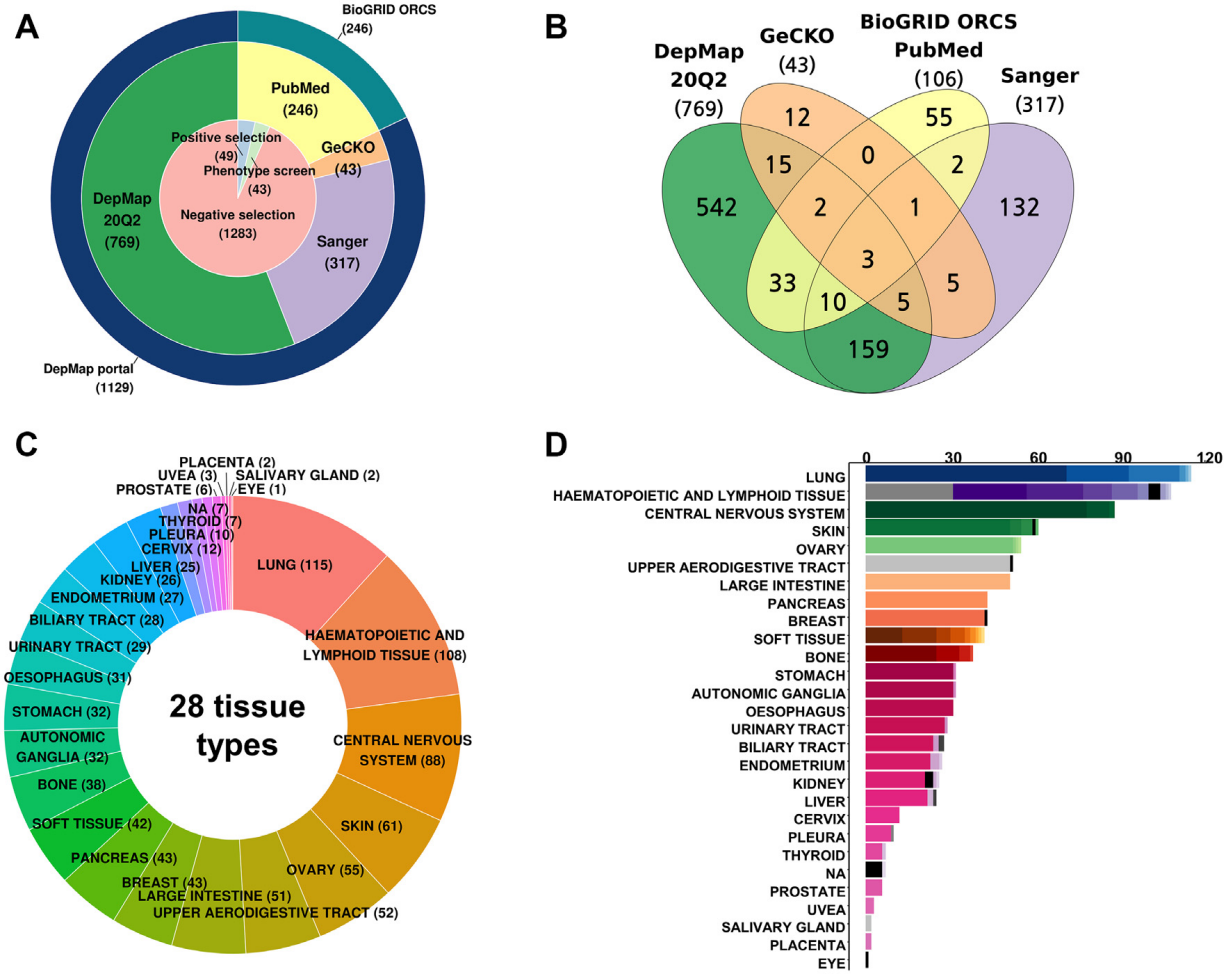
Statistics of iCSDB. (**A**) Number of screens according to the source database and screen type. (**B**) Venn diagram of cell lines according to the source database. (**C**) Cell line distribution across the tissue type. (**D**) Number of cell lines across tissue and cancer types. Different colors in the bar graph indicate different cancer types.

We have compiled two major sources of CRISPR-Cas9 screens: DepMap portal (Public 20Q2) and BioGRID ORCS (Ver. 1.0.4). DepMap portal itself is an integrated database of three large-scale projects (the Broad Achilles, Sanger and GeCKO), including 1129 screens across 921 cell lines (Figure 2A and B). BioGRID ORCS covered additional 246 screens across 106 cell lines from 62 PubMed articles (redundant entries with DepMap portal were excluded). Collectively, iCSDB included 1375 screens across 976 cell lines, majority of those being negative selections. Notably, positive or phenotypic screens were minor but non-negligible (∼6.7%). Thus, the PubMed collection reflects more diverse types of screening. We classified cell lines into 28 tissue types as provided by the CCLE consortium and 70 cancer types as defined in the NCI Thesaurus (Ver. 20.04d, release date 27 April 2020) (Figure 2C and D). Cellosaurus information on cell lines was utilized in the manual curation process (14).

Aggregation of data from multiple sources provides an opportunity to compare the results from different groups, which can be useful to confirm or validate observations in independent data, thus yielding reliable results. In our compendium dataset, we found that 246 cell lines were screened repeatedly, with 21 cell lines screened over four times. However, direct comparison is often difficult because of batch effects from different experimental conditions and analysis methods. We carefully examined the source of batch effects and found that CRISPR library and quantification algorithm were the major players. Of note, our compendium data set included 34 unique CRISPR libraries, where AVANA of Broad Achilles, Sanger and GeCKO libraries were used most frequently. Dempster *et al.* (8) examined the agreement between the Broad and Sanger CRISPR screens and concluded that two results are highly concordant (in terms of ranking) after removing batch effect using Combat in the sva R package (15).

**Figure 3.**
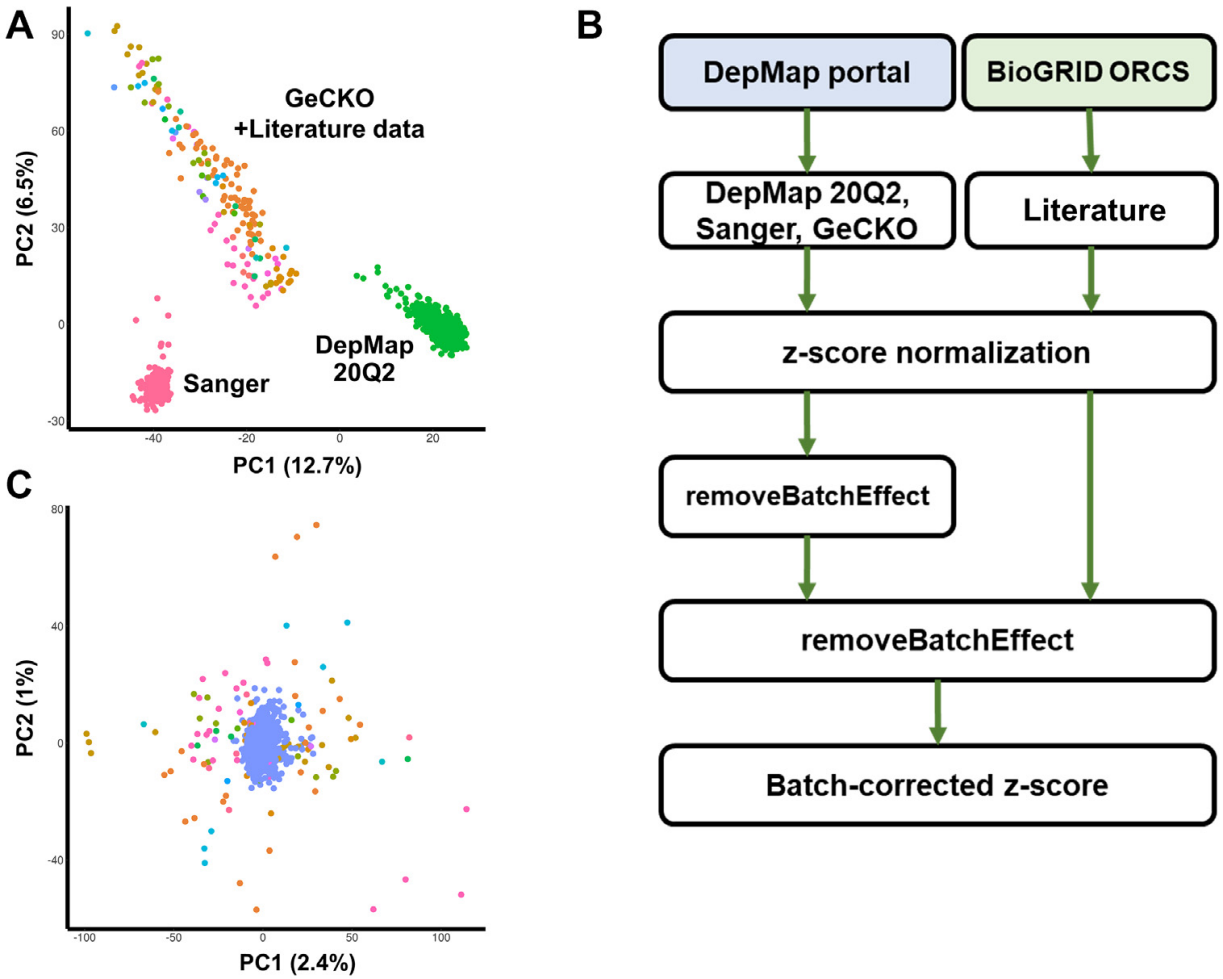
Batch effect in CRISPR screens. (**A**) Principal component analysis (PCA) plot after *z*-score normalization before batch correction. Each screen was colored according to the CRISPR library ID. Note that screens from PubMed were highly scattered and overlapped partially with the GeCKO library (in orange color). (**B**) Pipeline for correcting batch effects. (**C**) PCA plot after the two-step batch correction. Blue dots indicate screens from DepMap portal (DepMap 20Q2, Sanger, and GeCKO).

Merging data from PubMed articles make the comparison even more difficult because of diverse experimental setups and conditions. We converted all screen scores into the *z*-scores to obtain a single uniform scale. Nevertheless, the PCA analysis showed that batch effect was significant even within the DepMap portal dataset that reprocessed their whole data with CERES method accounting for the copy number-specific effect (16) (Figure 3A). Of note, we used removeBatchEffect function in the limma R package (v3.44.3) (17) instead of Combat mainly because removeBatchEffect allowed missing values that occurred mainly due to different number of target genes in different CRISPR libraries.

Neither removeBatchEffect nor Combat removed the batch effect successfully in a single step (Supplementary Figure S1A) probably because the variability in the PubMed and GeCKO data was higher than the one in DepMap or Sanger screen data (Figure 3A). Thus, we devised a two-step process for removing batch effects (Figure 3B). In the first step, batch correction was performed for screen results from the DepMap portal taking three different CRISPR libraries into account. The result was subsequently unified to include all screens from PubMed articles (Supplementary Figure S1B) and the second round of batch correction was carried out. This two-step process removed the batch effect successfully (Figure 3C). Screen results for BXPC3 cell line whose data are available in all four data sources were shown for example in Supplementary Figure S2. The correlation between different data sets increased substantially after our batch correction, thus demonstrating higher reliability in comparing multiple screen results.

### User interface and analytical tools

**Figure 4.**
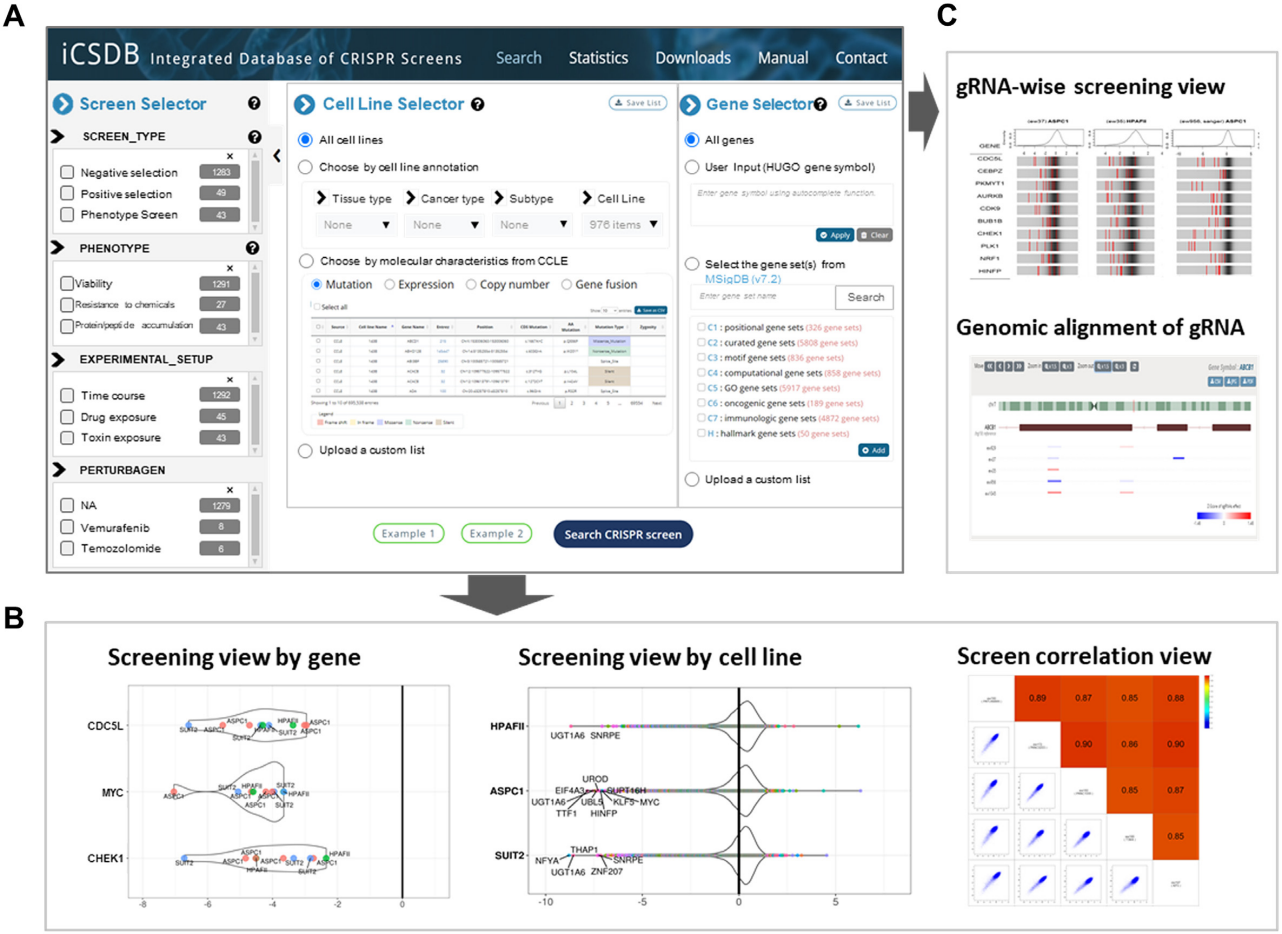
User interface of iCSDB. (**A**) Query interface for screen, cell line and gene selectors. (**B** and**C**) Visualization utilities include screening view by gene or by cell line, correlation view, guide RNA-wise view and genomic alignment of guide RNAs.

iCSDB supports user selection in three categories: screens, cell lines and genes (Figure 4A). The screen selector includes screen types, phenotype endpoints, experimental setup and perturbagens such as treated chemicals or mutated genes, following the annotation scheme in the BioGRID ORCS with minor modifications. Each entry comes with up-to-date number of database contents, where the most abundant one is negative selection of cell viability without any external exposure. The phenotype endpoint of all screens from DepMap portal was viability, but a variety of endpoints were assayed in the PubMed screens including resistance to chemicals, protein/peptide accumulation, toxin resistance, etc. Similarly, the experimental setup category included many different types of exposures (e.g. drug, ligand, toxin, etc.) from the PubMed screens. Thus, PubMed screens show diverse applications of CRISPR screening even though the number is rather small at this point.

Cell line selector allows users to choose cell lines according to the tissue type, cancer type and cancer subtype. Molecular characteristics may be used to select cell lines with specific mutation, expression, copy number or gene fusion event. For example, cell lines with *BRAF* mutations, or with AKL fusion genes, or with MET amplification might be useful to study the acquired resistance to targeted drugs. Gene selector is available when users want to examine only a subset of genes, which could be manually typed in or chosen from the gene sets in the MSigDB database (18).

Once the screens, cell lines and genes of interest are chosen, relevant CRISPR screen results can be explored in various visualization schemes. Screening view by gene or cell line shows the CRISPR screen score in a violin or box plot format that supports diverse interactive features such as balloon help, zooming, panning, etc. (Figure 4B). Correlation view allows users to compare two different screens. Finally, we devised a guide RNA (gRNA) level screen view that shows the knockout efficiency for each gRNA with background distribution (Figure 4C). Genomic alignment of gRNAs is also available. Of note, gRNA information is available for most libraries in the DepMap portal (i.e. DepMap 20Q2, Sanger and GeCKO) and part (∼48%) of screens from BioGRID ORCS PubMed.

It is often the case to browse the screen results in a categorical fashion. We support the browse function in the statistics page, where users may click any entry (or number) to choose the relevant cell lines and examine the screen result readily.

### A case study—screen on KRAS mutant cell lines

To demonstrate the utility of iCSDB, we compared the screen results for cell lines with KRAS mutations. KRAS is the most commonly mutated oncogene in human cancer including pancreatic, colon, lung and brain tumors. Numerous attempts to develop drugs targeting KRAS mutants directly were unsuccessful and targeting synthetic lethal vulnerabilities have been widely exploited. Several genome-scale LoF screens have been performed with siRNA, shRNA and CRISPR libraries (19).

**Figure 5.**
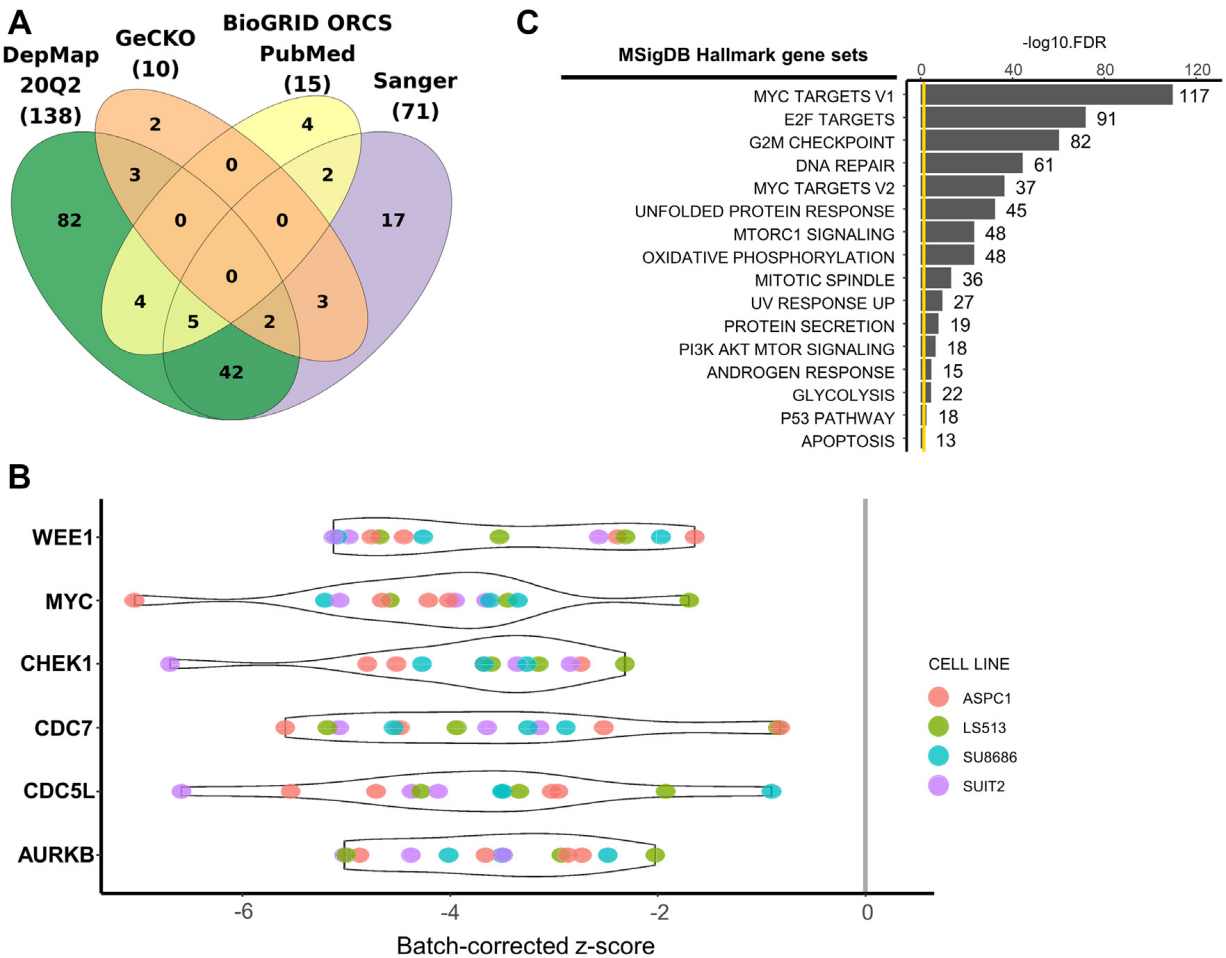
Synthetic lethal vulnerability screens for KRAS mutant cell lines. (**A**) Venn diagram of cell lines with KRAS mutations among different data sources. (**B**) CRISPR screen scores (batch corrected) for top 6 genes. Each screen was colored according to the cell line ID. (**C**) Enrichment score of Hallmark gene sets in MSigDB.

We identified 241 negative screens across 165 cell lines with KRAS mutations, 61 of those screened by multiple sources (Figure 5A). G12D mutation was the most common with 48 cell lines with 76 screens. We focused on four cell lines with G12D mutation used in three different sources (ASPC1, SUIT2, SU8686 from pancreatic cancer, and LS513 from colorectal cancer). We identified 15 screens from those four cell lines in iCSDB. Regarding genes within top 25% quartile of each screen as positive hits, we selected 1466 genes that were positive in >10 screens. The violin plots of screen scores are shown for six top-scoring genes (*WEE1*, *MYC*, *CHEK1*, *CDC7*, *CDC5L* and *AURKB*) (Figure 5B). Gene set analysis using the hallmark gene sets from MSigDB (18) showed significant association with the E2F-mediated MYC regulation that is a typical proliferation signal of epithelial cancer cells (20) (Figure 5C). DNA repair process and PI3K-AKT-mTOR signaling were also highlighted. All of these pathways are being intensely exploited for combination therapy targets recently (21). Of note, iCSDB includes other KRAS mutations—13 cell lines with G12V mutation, 8 cell lines with G12C mutation and 6 cell lines with G13D mutation. Comparing these data with G12D mutation may reveal context-specific combination therapies, which could be the next generation treatment of KRAS mutant cancers that are notorious for its heterogeneity.

## CONCLUSION

iCSDB is a meta-database to include human CRISPR screen data from the DepMap and BioGRID ORCS resources. As demonstrated in the case study, it provides an opportunity to identify drug targets to overcome resistance in cancer. Combining the screen data with the drug sensitivity data would open a systematic way for finding the mechanism-of-action of drugs (5) or for identifying new drug candidates by repurposing (22). To accommodate such needs, we plan to upgrade iCSDB to include the drug sensitivity data in the GDSC (4,23) and LINCS (24) databases. Regular update is essential in these rapidly evolving fields of genetic and drug screens. We plan to update our databases biannually to keep the contents up-to-date. iCSDB would be a useful resource for research communities in drug discovery as well as in basic molecular and cellular biology.

## Supplementary Material

gkaa989_Supplemental_Files
