# Supplementary material for: iCSDB: an integrated database of CRISPR screens
Source: Nucleic Acids Res. 2020 Nov 2;49(D1):D956–61. doi: 10.1093/nar/gkaa989 (PMC7779034; doi:10.1093/nar/gkaa989)
Supplement: gkaa989_Supplemental_Files [file gkaa989_supplemental_files.zip › Supplementary material legends.docx]

**Supplementary material legends**

**Supplementary Figure S1**. PCA plot of CRISPR screens. (A) After one-step batch correction using removeBatchEffect. The library colors follow the scheme in Figure 3A. (B) After the first step of two-step batch correction. Note that batch effect in the DepMap portal was successfully removed at this stage.

**Supplementary Figure S2**. Correlation plot of CRISPR screen results for BXPC3 cell line.
