# Supplementary figures and images for: iCSDB: an integrated database of CRISPR screens
Source: Nucleic Acids Res. 2020 Nov 2;49(D1):D956–61. doi: 10.1093/nar/gkaa989 (PMC7779034; doi:10.1093/nar/gkaa989)

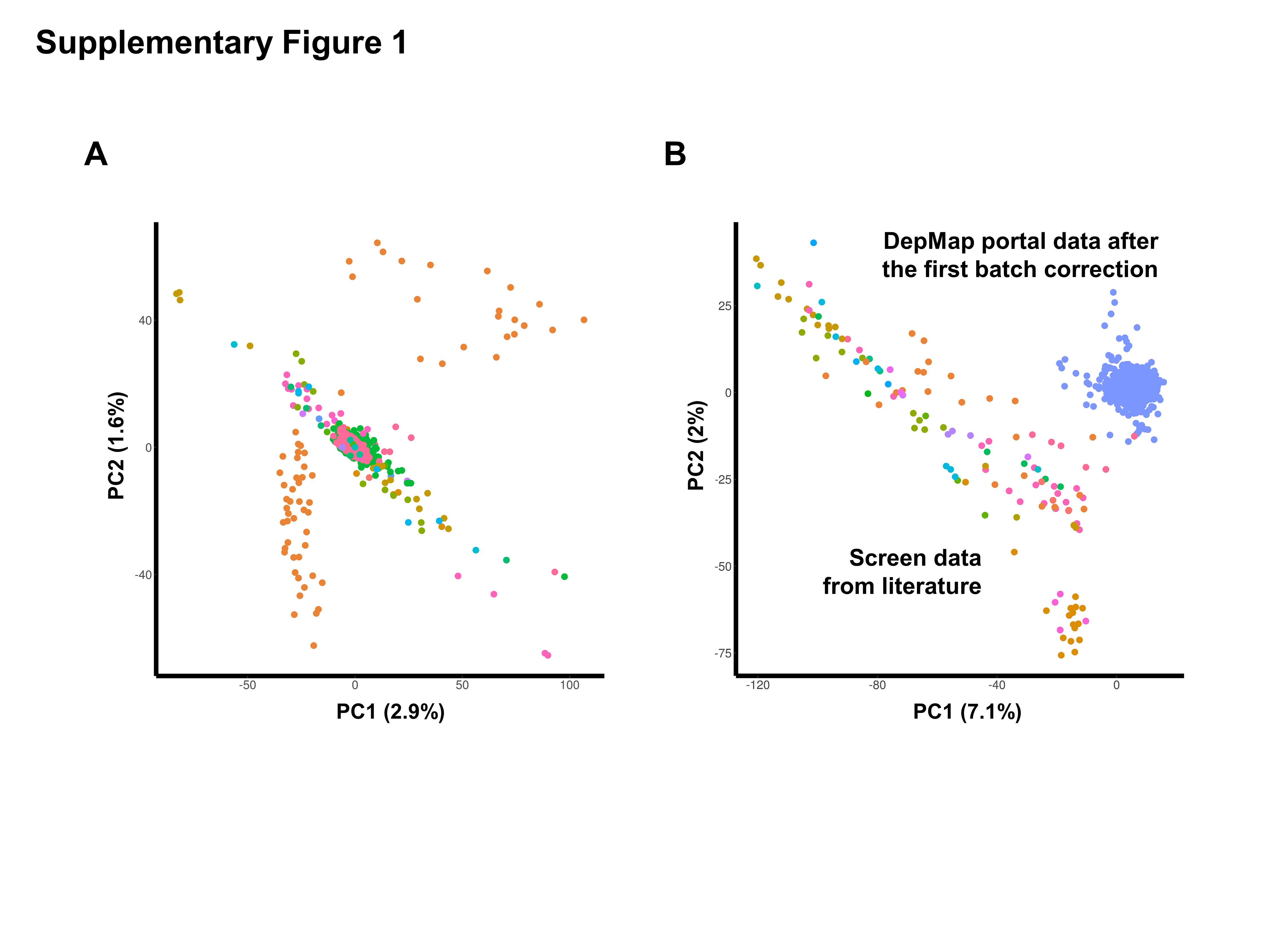

Supplement: gkaa989_Supplemental_Files [file gkaa989_supplemental_files.zip › Figure S1r.png]

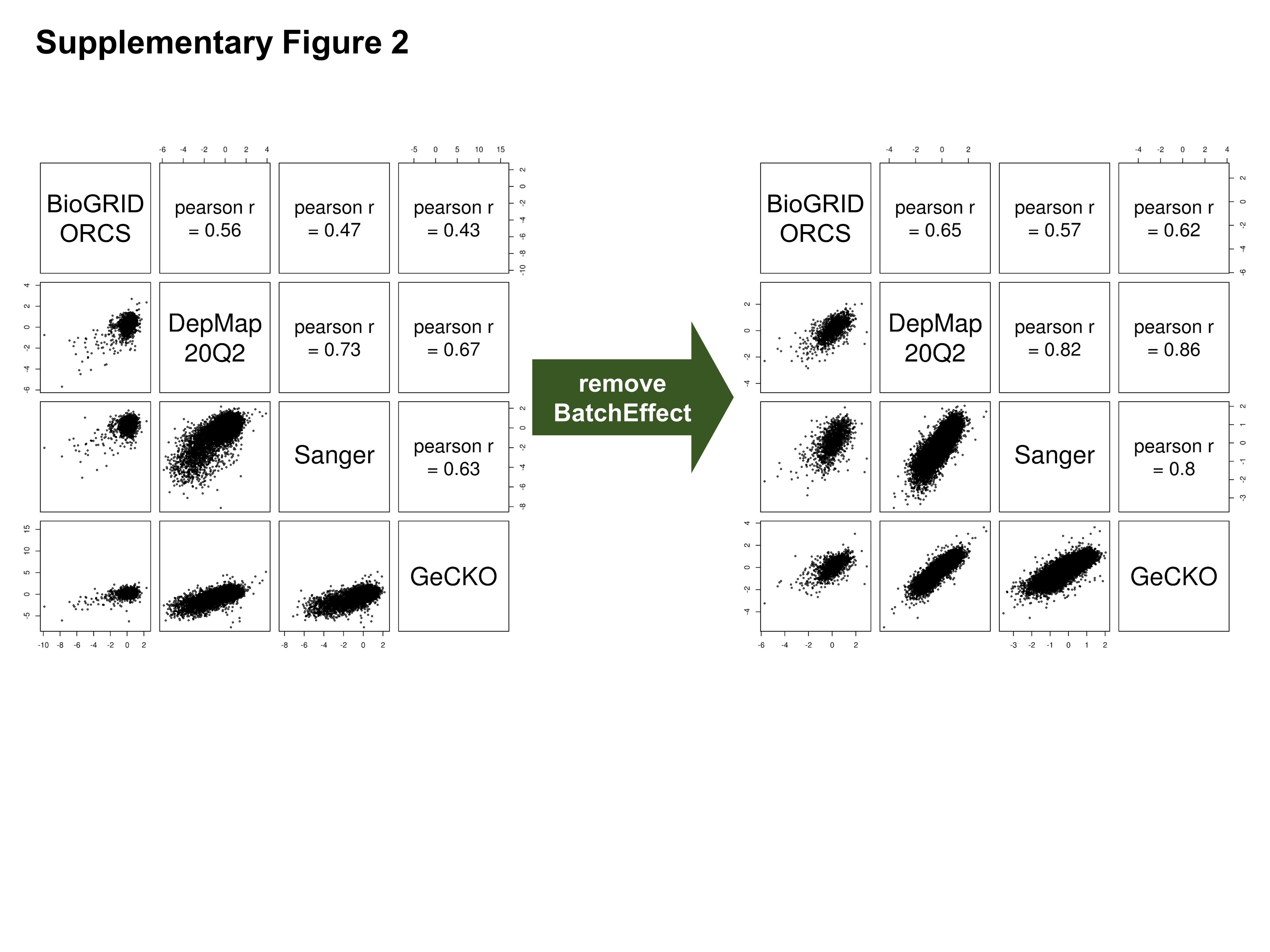

Supplement: gkaa989_Supplemental_Files [file gkaa989_supplemental_files.zip › Figure S2r.png]
